# *Krüppel*-Like Factor 5 Protects against Murine Colitis and Activates JAK-STAT Signaling *In Vivo*

**DOI:** 10.1371/journal.pone.0038338

**Published:** 2012-05-31

**Authors:** Marie-Pier Tetreault, Rami Alrabaa, Megan McGeehan, Jonathan P. Katz

**Affiliations:** Division of Gastroenterology, Department of Medicine, University of Pennsylvania Perelman School of Medicine, Philadelphia, Pennsylvania, United States of America; Charité, Campus Benjamin Franklin, Germany

## Abstract

Inflammatory bowel disease (IBD), which is characterized by chronic or recurring inflammation of the gastrointestinal tract, affects 1.4 million persons in the United States alone. KLF5, a *Krüppel*-like factor (KLF) family member, is expressed within the epithelia of the gastrointestinal tract and has been implicated in rapid cell proliferation, migration, and remodeling in a number of tissues. Given these functions, we hypothesized that constitutive *Klf5* expression would protect against the development of colitis *in vivo*. To examine the role of KLF5 *in vivo*, we used the *Villin* promoter to target *Klf5* to the entire horizontal axis of the small intestine and colon. *Villin-Klf5* transgenic mice were born at normal Mendelian ratios and appeared grossly normal to at least 1 year of age. Surprisingly, there were no significant changes in cell proliferation or in the differentiation of any of the intestinal lineages within the duodenum, jejunum, ileum, and colon of *Villin-Klf5* mice, compared to littermate controls. However, when *Villin-Klf5* mice were treated with dextran sodium sulfate (DSS) to induce colitis, they developed less colonic injury and significantly reduced disease activity scores than littermate controls. The mechanism for this decreased injury may come via JAK-STAT signaling, the activation of which was increased in colonic mucosa of DSS treated *Villin-Klf5* mice compared to controls. Thus, KLF5 and its downstream mediators may provide therapeutic targets and disease markers for IBD or other diseases characterized by injury and disruption of intestinal epithelia.

## Introduction

Intestinal epithelial cells typically form a selective permeability barrier to separate luminal contents from the underlying tissues [1]. This barrier is critical to protect the host against the numerous pathogens and other insults contained within the intestinal lumen. Disruption of the permeability barrier is seen in certain intestinal diseases such as inflammatory bowel disease (IBD), which affects 1.4 million persons in the United States [2], as well as celiac disease, ischemia, and intestinal infections [3], [4], [5]. IBD, which has two main forms, ulcerative colitis and Crohn's disease, is a chronic disease characterized by persistent or recurring inflammation and immune response within the gastrointestinal mucosa [2]. Thus, most treatments for IBD target the immune response. Yet, the ability of the intestine to repair itself following acute, chronic, or relapsing injury is also likely to be key for disease activity and progression.

Following mucosal injury, the initial response is of the intestine is epithelial restitution, with rapid migration of cells from the wound edge to restore surface epithelial continuity [5], [6]. Epithelial cells then undergo increased proliferation and cell differentiation to fully reconstitute the intestinal epithelium. Thus factors that upregulate cell proliferation and migration are likely to be critical for intestinal repair following inflammation and injury. A number of regulatory pathways have been shown to be important in intestinal injury, including the JAK-STAT pathways [1], [3], [6]. In mammals, the JAK/STAT pathway is a key signaling mechanism for growth factors and cytokines [7], and activation of STAT3, in particular, protects against the development of colitis and promotes intestinal repair following mucosal injury [8], [9], [10], [11], [12], [13].

The zinc-finger transcription factor *Krüppel*-like factor 5 (KLF5; also known as BTEB2) has been implicated in tissue repair, including in the intestine [14], [15]. KLF5 has an epithelial-specific expression pattern in the intestine and is localized primarily to the proliferating cells within the small intestinal crypts and the lower third of colonic crypts [16], [17]. KLF5 promotes rapid cell proliferation, migration, and remodeling [18], [19], [20], [21], [22], and mice with hemizygous deletion of Klf5 have greater sensitivity to dextran sodium sulfate (DSS) colitis than wild-type mice [23]. However, KLF5 has also been suggested to be an oncogene in the intestine [18], [24], [25] and thus the potential relevance of KLF5 activation in intestinal injury and repair is not certain. Interestingly, when *Klf5* was targeted to esophageal epithelia in mice, we found no changes in the gross morphology and histology of the epithelium and no obvious increase in malignant potential, despite a clear increase in basal cell proliferation [26]. However, to date, the effect of increased KLF5 in intestinal epithelia *in vivo* has not been examined, and a protective effect of constitutive intestinal KLF5 expression on colitis has not yet been demonstrated.

Here, using the *Villin* promoter, we targeted *Klf5* throughout the entire intestinal epithelium, from duodenum to colon, along the entire crypt-villus axis, and throughout the colonic crypts. We then treated *Villin-Klf5* transgenic mice with DSS to induce colitis and examine the effect of KLF5 on intestinal injury and restitution *in vivo*.

## Materials and Methods

### Generation of Villin-Klf5 mice

All animal studies were approved by the Institutional Animal Care and Use Committee (IACUC) at the University of Pennsylvania. To express *Klf5* in intestinal epithelia, we cloned the complete coding sequence of murine *Klf5* [17] into the p12.4KVill plasmid (gift of Dr. Deborah Gumucio, University of Michigan) [27]. This construct was sequenced, and the *Villin-Klf5* fragment was excised for injection. Derivation of transgenic mice was accomplished by the University of Pennsylvania Transgenic and Chimeric Mouse Facility. We documented transgene integration in nine *Villin-Klf5* founder lines by PCR for the *Villin* promoter. Offspring were then screened for transgene expression by RNAse protection assays, Western blot, and immunohistochemistry, as described below, and a total of four lines demonstrated transgene expression. Since initial phenotypic analyses revealed no differences among the lines, further studies were carried out on a single *Villin-Klf5* transgenic line. Mice were backcrossed to C57BL/6 (Charles River Laboratories, Wilmington, MA) for at least 10 generations.

### DSS-induced acute injury

Two month-old control and *Villin-Klf5* mice were given 3.5% DSS (MP Biomedicals, Aurora, OH) in their drinking water for 7 days, whereas untreated mice received water alone. Mice were monitored daily for weight loss and visible signs of rectal bleeding. Occult bleeding was evaluated (Hemoccult; Beckmann Coulter, Fullerton, CA) at day 7, and disease index was calculated by assessing weight loss, occult blood, and stool consistency, as previously described [28]. Mice were sacrificed 0, 3, and 7 days after initiation of DSS treatment.

**Figure 1:**
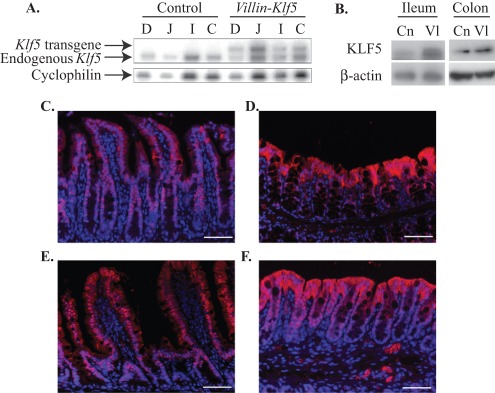
*Villin-Klf5* transgene was expressed in small and large intestinal epithelia. (**A**) RNAse protection assay demonstrated strong transgene expression in the duodenum (D), jejunum (J), ileum (I), and colon (C) of *Villin-Klf5* mice, compared to their littermate controls. *Cyclophilin* was used as a loading control. (**B**) At 1 month of age, compared to controls (Cn), *Villin-Klf5* mice (Vl) had increased KLF5 expression in the small intestine and colon by Western blot. (**C–D**) Immunofluorescence of 6 month-old control mice revealed nuclear KLF5 staining restricted to small intestinal crypts **(C)**, with weak nuclear staining in colonic crypt cells (**D**). (**E-D**) *Villin-Klf5* mice at 6 months of age had stronger KLF5 staining, with nuclear staining seen along the entire crypt-villus axis of the small intestine (**E**) and increased numbers of KLF5-positive cells in colonic mucosa (**F**). In both control and *Villin-Klf5* mice, cytoplasmic KLF5 staining was seen in the surface epithelia. Scale bars: 50 µm.

### Histology

*Villin-Klf5* mice and littermate controls were injected with 5-bromo-2-deoxyuridine (BrdU) Labeling Reagent (Life Technologies, Grand Island, NY) 60 min prior to sacrifice. For untreated mice, small and large intestines were removed at 1, 3, 6, and 12 months of age, examined grossly, and then processed for histology as previously described [26]. Briefly, the small and large intestine were dissected out, longitudinally splayed, flushed with ice-cold PBS, swiss-rolled, and fixed in 4% paraformaldehyde overnight at 4°C. Tissues were then embedded in paraffin, and 5-µm sections were applied to Probe-on Plus slides (Fisher Scientific, Pittsburgh, PA). Sample slides were stained with hematoxylin and eosin. For Alcian blue staining, 3% aqueous acetic acid was applied to the slides followed by addition of 1% Alcian blue in 3% acetic acid, pH 2.5, then counterstained with 0.1% nuclear fast red. Images were captured on a Nikon Eclipse E600 microscope (Nikon Instruments, Melville, NY) with a Photometrics CoolSNAP charge-coupled device camera (Roper Scientific, Tucson, AZ).

### RNA analyses

Total RNA was extracted from mouse intestine using the RNeasy Mini Kit (Qiagen, Valencia, CA) following manufacturer's instructions. Ribonuclease (RNase) protection assays were performed as described previously using 1 µg of total RNA per sample [26]. Probes were designed to span the 3′ end of the *Villin-Klf5* transgene, from the *Klf5* cDNA to just upstream of the polyadenylation signal, to protect fragments of 345 bp for endogenous *Klf5* and 459 bp for the *Villin-Klf5* transgene. A 103-nt cyclophilin probe was employed as an internal standard. RNA fragments obtained were separated on a Novex 6% TBE-urea acrylamide gel (Life Technologies, Carlsbad, CA) and the radioactive bands visualized on a Storm 840 phosphorimager (GE Healthcare Bio-Sciences, Piscataway, New Jersey). Quantitative real-time PCR analysis was performed on an ABI Step One Plus sequence detection system (Life Technologies) using conditions and primer concentrations suggested by the SyBr Green PCR master mix (Life Technologies) protocol. Reverse transcription was performed with Maxima® First Strand cDNA synthesis kit for RT-qPCR (Thermo Fisher Scientific). The TATA box binding protein (TBP) or Glyceraldehyde-3-phosphate dehydrogenase (GAPDH) genes were used as the internal control. Primer sequences are available upon request.

### Immunohistochemistry and quantitation of proliferative cells

We performed microwave antigen retrieval and processed the tissues as previously described [26], followed by an incubation with one of the following primary antibodies: 1∶5,000 anti-KLF5, which we generated previously [29]; 1∶15,000 rat anti-BrdU (Accurate Chemicals, Westbury, NY); 1∶1,500 Ki-67 (Vector Laboratories, Burlingame, CA); 1∶5,000 Lysozyme (Dako, Carpinteria, CA); or 1∶50 rabbit anti-phospho-Stat3 (Y705) (Cell Signaling Technology Inc., Beverly, MA). Species-specific secondary antibodies were added, and antibody binding was detected as previously described [26]. For fluorescent labeling, 1∶600 Cy3 (Jackson ImmunoResearch, West Grove, PA) antibody was used. Alkaline phosphatase activity was analyzed by incubation with 5-bromo-4-chloro-3-indolyl-phosphatase-4-nitro blue tetrazolium chloride. The proliferative index was determined by counting the numbers of BrdU- or Ki-67-labeled cells per crypt in a least five distinct regions of intestine from at least five *Villin-Klf5* mice and five littermate controls at each time point. Results were expressed as the average number of labeled cells ± SEM.

### Western Blots

Protein aliquots (20 µg from mouse tissue or cell lysates were separated by SDS-PAGE on 4–10% gels and electrotransfered onto PVDF membranes (EMD Millipore, Billerica, MA). Membranes were blocked in PBS containing 5% powdered milk and 0.05% Tween-20% for 1 hour at 25°C then incubated overnight at 4°C with primary antibody in blocking solution followed by horseradish peroxidase-conjugated secondary antibody (1∶10,000) for 1 hour. Blots were visualized using the Immobilon ECL system (EMD Millipore). Protein concentrations were assayed using BCA Protein Assay Reagent (Thermo Fisher Scientific) with BSA as standard.

### Wounding assay

Cells were wounded using the scratch assay technique [30]. IEC-6 cells, transduced with pFB-neo or pFB-*Klf5* retrovirus [26], were seeded in 6-well plates and allowed to reach confluence, after which the functional epithelial monolayers were wounded linearly several times. Cells were harvested and then lysed in Triton X-100 sample buffer for use in Western blotting.

## Results

To evaluate the consequences of KLF5 overexpression in intestinal epithelial cells *in vivo*, we used the 12.4 Kb *Villin* promoter [27] to target *Klf5* along the entire horizontal and longitudinal axes of the intestine in mice. *Villin-Klf5* mice were born at the appropriate Mendelian ratio and appeared grossly normal up to at least 12 months of age. Transgene expression throughout the small and large intestine of *Villin-Klf5* mice was confirmed by RNAse protection assay (Figure 1A). Increased KLF5 protein levels were demonstrated by Western blot (Figure 1B) of small intestine and colon from *Villin-Klf5* mice compared to littermate controls. In control mice, KLF5 was restricted to the small intestinal crypts (Figure 1C) and the lower third of colonic crypts (Figure 1D), regions of active cell proliferation, as demonstrated by immunofluorescence. In contrast, KLF5 expression in *Villin-Klf5* mice was seen in both the small intestinal crypts and villi (Figure 1E) and throughout colonic crypts (Figure 1F).

**Figure 2:**
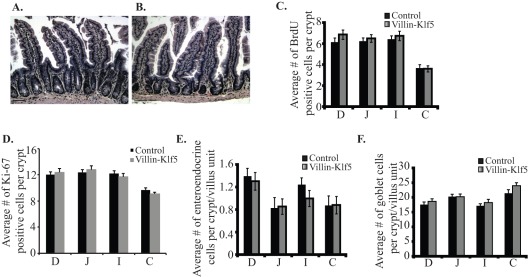
*Villin-Klf5* transgenic mice had normal-appearing intestinal mucosa. (**A–B**) Compared with 1 year-old littermate controls (**A**), hematoxylin and eosin (H&E)-stained ileal epithelia, along with underlying lamina propria and muscular layers, appeared normal in 1 year-old *Villin-Klf5* mice (**B**). Findings were similar in duodeum, jejunum, and colon of *Villin-Klf5* mice. Scale bars: 50 µm. (**C–D**) Quantitation of BrdU-labeled (**C**) or Ki-67 positive (**D**) cells revealed no changes in proliferation in the small intestine or colon of *Villin-Klf5* mice at 1 year of age, compared to littermate controls. (**E**) The number of chromogranin-A positive enteroendocrine cells per crypt-villus unit was unchanged between control and *Villin-Klf5* mice at 1 year of age. (**F**) By Alcian blue staining, no difference was observed in the number of goblet cells within small intestine or colon of 1 year-old control and *Villin-Klf5* mice. Similar results were observed for proliferation and differentiation of intestinal cell types in 1 month-, 3 month-, and 6 month-old mice. D =  Duodenum, J = Jejunum, I = Ileum, C = Colon.

Compared to littermate controls (Figure 2A), *Villin-Klf5* mice had no morphological changes in the small intestine (Figure 2B) or colon (not shown) at 12 months of age. Moreover, there was no evidence of dysplasia, polyp formation, or cancer up to at least 12 months of age (not shown). Given our recent demonstration of KLF5 as a positive regulator of proliferation in esophageal epithelia of mice [26], we examined whether intestinal epithelial proliferation was similarly altered in *Villin-Klf5* mice. Surprisingly, we found no significant difference in the number of proliferating cells between control and *Villin-Klf5* mice in any intestinal segment (Figure 2C–D); proliferating cells were restricted to the small intestinal crypts and the base of the colonic crypts in both control and *Villin-Klf5* mice (not shown). We next examined if intestinal cell differentiation and lineage specification were altered by transgenic expression of *Klf5*. Numbers of enteroendocrine cells, as determined by staining for chromagranin A (Figure 2E), were unchanged in small and large intestines of *Villin-Klf5* mice, compared to littermate controls. Likewise, goblet cells, visualized by Alcian Blue staining, were seen at similar numbers in control and *Villin-Klf5* mice (Figure 2F). Paneth cell and enterocyte differentiation was also unaffected by increased expression of *Klf5* in *Villin-Klf5* mice (not shown). Thus, at baseline, increased *Klf5* expression in intestinal epithelia is not sufficient to perturb normal epithelial homeostasis *in vivo*.

**Figure 3:**
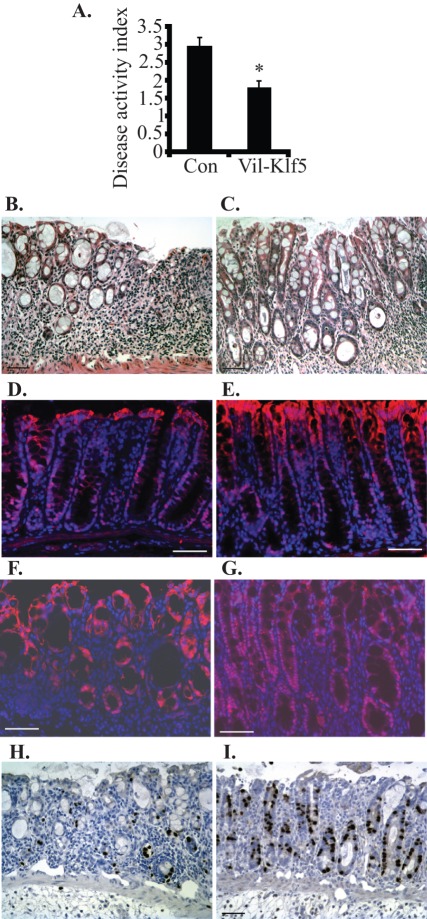
*Villin-Klf5* mice are less susceptible to injury following DSS treatment. (**A–G**) Eight-week-old mice were treated with 3.5% DSS for 7 days. (**A**) Compared to littermate controls (Con), *Villin-Klf5* mice (Vil-Klf5) had decreased sensitivity to experimental colitis with DSS, as assessed by disease activity index (*p = 0.005; n = 6 pairs). (**B, C**) DSS-treated control mice (**B**) had reduced intestinal epithelial repair and increased susceptibility to colitis, compared to *Villin-Klf5* mice (**C**). Note the preservation of crypt architecture in *Villin-Klf5* mice. (**D–G**) Immunofluorescence confirmed that, at day 0, compared to controls (**D**)**,** *Villin-Klf5* mice (**E**) had increased KLF5 expression in colonic epithelial cells. At day 7 of DSS treatment, compared to controls (**F**)**,** nuclear KLF5 expression in colonic epithelia of *Villin-Klf5* mice was markedly increased (**G**). (**H, I**) Compared to colonic mucosa from control mice (**H**), colonic mucosa of *Villin-Klf5* mice (**I**) demonstrated increased numbers of BrdU-positive cells adjacent to the sites of ulceration. Scale bars: 50 µm.

KLF5 is known to control cell migration, wound repair, and inflammatory responses [20], [23], [31]. Therefore, we examined whether increased *Klf5* expression protects intestinal epithelia from tissue damage. *Villin-Klf5* and control mice were challenged with 3.5% DSS for 7 days to induce experimental colitis. Disease activity index was determined by scoring changes in weight, hemoccult positivity, and stool consistency [28] and was significantly lower in *Villin-Klf5* mice than littermate controls (Figure 3A). Compared to colonic tissues from control mice (Figure 3B), colonic sections from *Villin-Klf5* mice (Figure 3C) revealed less tissue damage and improved regeneration of the intestinal mucosa following DSS-induced injury. Notably, crypt architectural distortion and focal thinning of colonic surface epithelia were observed in control mice, while crypt architecture was better preserved in the regenerating colonic mucosa of *Villin-Klf5* mice (Figure 3C). Higher numbers of surface epithelial erosions were also observed in the colon of control mice treated with DSS, compared to *Villin-Klf5* mice (not shown). At day 0 of DSS treatment, compared to controls (Figure 3D), nuclear KLF5 expression was increased in colonic mucosa of *Villin-Klf5* mice (Figure 3E). KLF5 was also expressed in colonic epithelia of control mice following DSS treatment (Figure 3F), but *Villin-Klf5* mice (Figure 3G) had substantially more KLF5 in the epithelial cells of the regenerating intestinal mucosa adjacent to the regions of ulceration. Moreover, expression was clearly nuclear in the lower half of the crypts of *Villin-Klf5* mice, consistent with the established role of KLF5 as a transcriptional regulator. To characterize changes in proliferation in *Villin-Klf5* mice challenged with DSS, we pulse-labeled colonic epithelial cells with BrdU. Compared to controls (Figure 3H), *Villin-Klf5* mice (Figure 3I) had more proliferating cells by BrdU labeling in areas of intestinal epithelial regeneration. Thus, *Klf5* overexpression, while not altering colonic homeostasis in the unchallenged state, protects against colonic injury and promotes epithelial restitution following challenge with DSS.

**Figure 4:**
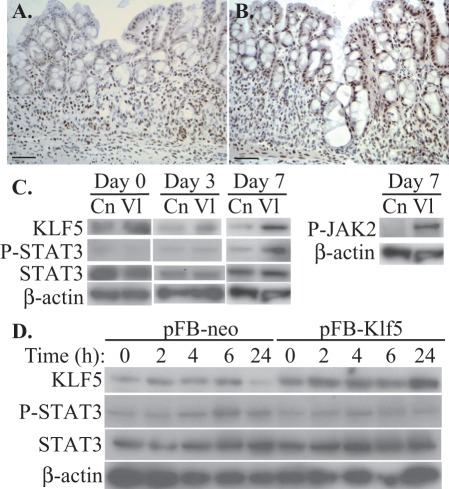
*Klf5* overexpression in intestinal epithelia increases STAT3 phosphorylation *in vivo*. (**A, B**) Immunohistochemistry of colonic mucosa from control (**A**) and *Villin-Klf5* (**B**) mice following DSS treatment revealed more phosphorylated STAT3 in regenerating colonic epithelial cells from *Villin-Klf5* mice. (**C**) Western blot of colonic epithelial scrapings from control (Cn) and *Villin-Klf5* (Vl) mice at the indicated time points following DSS treatment revealed increased phospho-STAT3 and phospho-JAK2 in *Villin-Klf5* mice at day 7, while phospho-STAT3 was decreased in *Villin-Klf5* mice at day 0 and unchanged at day 3. (**D**) Western blots confirmed increased KLF5 expression in IEC-6 cells transduced with *pFB-Klf5* compared to pFB-neo. However, following linear wounding, no induction of phosphorylated STAT3 was observed in *pFB-Klf5* infected cells, compared to control pFB-neo infected cells.

KLF5 can regulate cytokine production in response to injury or other stimuli [32], [33], [34], and multiple cytokine-induced signaling pathways converge on the key transcription factor STAT3 [35]. Moreover, activated STAT3 is critical both for protection against colitis and for the restoration of intestinal integrity during colitis [8], [9], [10], [11], [12]. We hypothesized that the protective effect of KLF5 following intestinal mucosal injury by DSS was mediated by activation of the JAK/STAT pathway. To assess the role of STAT3 activation, we initially performed immunohistochemistry for phosphorylated STAT3 in control and *Villin-Klf5* mice following DSS treatment. In control mice treated with DSS, phosphorylated STAT3 was present in intestinal epithelial cells and in the stroma (Figure 4A), but levels of phosphorylated STAT3 were much higher in regenerating epithelial cells of *Villin-Klf5* mice (Figure 4B). These findings were confirmed by Western blotting of colonic epithelial scrapings from mice treated with DSS, and this increase in STAT3 activity in *Villin-Klf5* mice also correlated with elevated phosphorylated JAK2 (Figure 4C). Of note, no significant changes in STAT3 phosphorylation levels were observed between control and *Villin-Klf5* mice at days 0 and 3 of DSS treatment. These data suggest that KLF5 may regulate intestinal epithelial regeneration in experimental colitis through the JAK2/STAT3 pathway.

**Figure 5:**
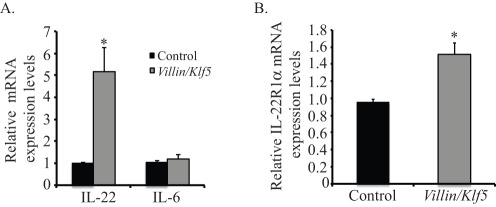
*Klf5* overexpression increases IL-22 signaling in the colonic microenvironment following DSS treatment. (**A**) By quantitative real-time PCR, *Villin-Klf5* mice had increased *IL-22* mRNA expression in the colonic stromal compartment after 7 days of DSS treatment, compared to controls, while *IL-6* mRNA expression was unchanged. (**B**) Quantitative real-time PCR of epithelial scrapings from control and *Villin-Klf5* mice treated for 7 days with DSS showed increased *IL-22R1α* expression in the epithelia of *Villin-Klf5* mice.

We next sought to determine how KLF5 regulates STAT3 activation in the context of mucosal injury. IEC-6 intestinal epithelial crypt cells were retrovirally transduced with pFB-neo or *pFB-Klf5*, and confluent monolayers were injured and examined at different time points following wounding. Western blot analyses showed that phospho-STAT3 levels increased slightly following wounding compared to unstimulated cells (Figure 4D), consistent with previous findings that STAT3 plays a role in epithelial cell migration *in vitro* [10]. Surprisingly, however, *Klf5* overexpression did not increase STAT3 phosphorylation compared to controls. Thus, the mechanism of STAT3 activation by KLF5 in the context of colonic mucosal wound healing appears to be non cell-autonomous.

STAT3 activation in murine colitis is dependent on IL-22, which is secreted by cells of the innate immune system [12]. To determine whether IL-22 signaling was increased in *Villin-Klf5* mice, we analyzed *IL-22* and *IL-6* mRNA expression in lysates of colonic stroma following removal of the epithelium. Compared to control mice, *Villin-Klf5* mice had significantly increased *IL-22* expression, whereas *IL-6* expression was unchanged (Figure 5A). Consistent with these findings, *Villin-Klf5* mice demonstrated increased expression of *IL-22 receptor* mRNA in colonic epithelia, compared to control mice (Figure 5B).

**Figure 6:**
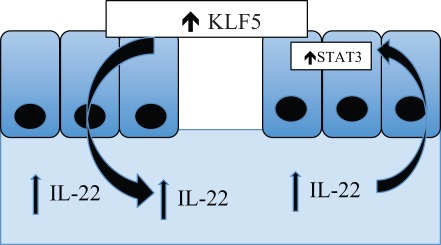
Model for the protective role of KLF5 in intestinal wound healing during colitis. KLF5 induces the expression of specific cytokines by epithelial cells, leading to the recruitment of immune cells and elaboration of IL22, which then activates STAT3 signaling within the colonic epithelial cells.

## Discussion

Maintenance of intestinal epithelial homeostasis depends on a careful balance between cell proliferation, differentiation, migration, and apoptosis [36], [37]. Moreover, the presence of a functional mucosal barrier is an essential for protection against numerous intestinal diseases [38]. Numerous studies have illustrated the importance of the *Krüppel*-like factor family member KLF5 in the regulation of epithelial homeostasis and diseases [15]. Recently, mice with hemizygous deletion of *Klf5* were found to have greater sensitivity to DSS colitis than wild-type mice [23]. Yet the consequences of increased KLF5 expression in the intestine have not been examined *in vivo*.

A large body of evidence implicates KLF5 as a positive regulator of proliferation, including in non-transformed intestinal epithelial cells *in vitro* [18], [39], [40]. Moreover, transgenic expression of *Klf5* in murine esophageal epithelia *in vivo* results in increased basal cell proliferation [26]. Surprisingly, transgenic expression of *Klf5* did not significantly alter intestinal morphology, cell proliferation, or cell lineage allocation, suggesting that endogenous levels of KLF5 are sufficient to control these processes. Interestingly, KLF5 has also been reported to have oncogenic properties and to mediate transformation by K-Ras during intestinal tumorigenesis [24]. Yet, no dysplasia, polyps, or cancers developed in *Villin-Klf5* mice up to at least 12 months of age, suggesting that KLF5 alone is not sufficient to induce cancer in the intestine. Thus, context is important for KLF5 function, and combined with our recent finding that KLF5 loss in the context of p53 ablation drives invasive progression of squamous cell cancer [41], it would be interesting to examine whether similar cooperativity exists in the intestine.

Patients with inflammatory bowel disease (IBD) show evidence of intestinal mucosal barrier disruption, prolonged inflammatory response, and severe mucosal lesions [42]. Therefore, a better understanding of the pathways involved in the regeneration of the intestinal mucosa following injury is crucial. Several lines of evidence suggest that KLF5 plays a role in response to injuries. KLF5 is an important regulator of cardiovascular remodeling [21], and keratinocyte migration [20], and mice with *Klf5* haploinsufficiency exhibit greater sensitivity to DSS-induced colonic injury [23] but are protected from renal injuries [34]. Taken together, these observations show the importance of studying the role of KLF5 in different cell types, tissues, and context. The results presented here demonstrate that epithelial-specific expression of KLF5 protects against the development of colitis *in vivo*. The mechanism for this resistance to colitis may be attributable to increased IL-22/JAK2/STAT3 signaling, since recent studies indicate that activation of STAT3 within the intestinal epithelium plays an important role in mucosal tissue repair through secretion of IL-22 from innate immune cells [8], [9], [10], [11], [12]. Moreover, JAK/STAT signaling has been implicated in intestinal homeostasis by regulating proliferation of intestinal stem cells [43], raising the possibility that KLF5 induction could play a role in upregulating intestinal stem cell proliferation following injury. Following DSS treatment, we observed increased intestinal epithelial cell proliferation and localized STAT3 activation; this STAT3 activation was confined to crypts adjacent to the wounded areas. These results correlated with an increase in *IL-22* mRNA levels in the stroma and increased IL-22R in the intestinal epithelia of *Villin-Klf5* mice.

STAT3 activation can be triggered by numerous cytokines [44], and the context of STAT3 activation for inflammation is particularly important. For example, STAT3 activation in intestinal epithelial cells protects against the development of colitis and enhances intestinal restitution, findings consistent with our observations [10], [12], [45]. STAT3 deletion in macrophages and neutrophils produces chronic enterocolitis [46], while STAT3 expression in T cells is essential for the induction of colitis [47]. In contrast to our findings, STAT3 activation was reported to promote colitis and prevent wound healing [48], but this study does not discriminate in which cell-type STAT3 is hyperactivated. In aggregate, these studies highlight a cell-type specific role of STAT3 in the regulation of mucosal healing following colitis. Interestingly, STAT3 activation in enterocytes may also increase the development of colitis-associated cancer [45]; thus the long-term effects of KLF5-mediated STAT3 induction in colitis merit further study.

Our results also demonstrate that STAT3 activation by KLF5 following DSS treatment is non cell-autonomous. We propose a model in which KLF5 protects following colonic injury through secretion of IL-22 by infiltrating immune cells and subsequent activation of STAT3 signaling (Figure 6). KLF5 can stimulate the expression of several different cytokines and chemokines [33], [34], and we propose that increased KLF5 expression in intestinal epithelial cells leads to the induction and elaboration of specific chemokines by these cells. In turn, these chemokines recruit innate immune cells secreting IL-22, and IL-22 binds to the IL-22 receptor to activate STAT3 within intestinal epithelial cells. The ability to augment these intestinal repair mechanisms may lead to improved treatments for diseases characterized by injuries to the intestinal epithelium. However, additional studies will be needed to identify the responsible chemokines and better define the link between KLF5 and IL-22/JAK/STAT3 signaling.

In sum, we have used a novel transgenic mouse model to demonstrate an essential role for KLF5 in intestinal epithelial homeostasis and in mucosal healing following induction of experimental colitis. Following DSS treatment, *Klf5* overexpression is associated with activation of STAT3 signaling *in vivo*. Importantly, there are no obvious consequences of *Klf5* overexpression on intestinal homeostasis in untreated mice up to 12 months of age. Thus KLF5 and STAT3 signaling provide important areas for further study in colitis and represent potential diagnostic and therapeutic targets for IBD and other diseases characterized by injury and disruption of intestinal epithelia.
